# Ethnic and biological differences in the association between physical activity and survival after breast cancer

**DOI:** 10.1038/s41523-020-00194-5

**Published:** 2020-10-09

**Authors:** Yunfeng Cao, Kathy B. Baumgartner, Kala Visvanathan, Stephanie D. Boone, Richard N. Baumgartner, Avonne E. Connor

**Affiliations:** 1 Department of Epidemiology, Johns Hopkins Bloomberg School of Public Health, Baltimore, MD USA; 2 Department of Epidemiology and Population Health and the James Graham Brown Cancer Center, University of Louisville, Louisville, KY USA; 3 The Sidney Kimmel Comprehensive Cancer Center at Johns Hopkins, Baltimore, MD USA

**Keywords:** Risk factors, Cancer epidemiology

## Abstract

Physical activity is recommended for most cancer patients as a nonpharmacological therapy to improve prognosis. Few studies have investigated the association between physical activity and breast cancer prognosis by ethnicity, biological, and modifiable risk factors for mortality. We investigated the association between physical activity and long-term survival among breast cancer survivors. A total of 397 survivors (96 Hispanic and 301 non-Hispanic White (NHW)) from the New Mexico HEAL study contributed baseline and biological data approximately 6 months after diagnosis. Study outcomes included all-cause, breast cancer-specific, and non-breast cancer mortality. The exposure was self-reported physical activity within the past month. Multivariable hazard ratios (HRs) and 95% confidence intervals (CIs) were estimated using Cox Proportional Hazards regression. A total of 133 deaths (53 breast cancer-specific deaths) were observed after a median follow-up time of 13 years. Engaging in >6.9 metabolic equivalent hours/week (MET-h/week) of moderate to vigorous physical activity (active) was inversely associated with all-cause mortality among all women (HR 0.66, 95% CI 0.43–0.99) and NHWs (HR 0.58, 95% CI 0.36–0.94). Active NHW women also had a reduced risk of non-breast cancer mortality (HR 0.56, 95% CI 0.31–0.99), compared to inactive women (0 MET-h/week). In subgroups, we observed the inverse associations with all-cause mortality among women >58 years old (*p*-interaction= 0.03) and with localized stage (*p*-interaction = 0.046). Our results confirm the protective association between physical activity and mortality after breast cancer diagnosis, and demonstrate that this association significantly differs by age and cancer stage. Larger studies are warranted to substantiate our findings.

## Introduction

Breast cancer is the most commonly diagnosed cancer in women in the United States and worldwide. Though breast cancer survivorship has been significantly prolonged with advanced screening and treatment technologies, breast cancer remains among the top common causes of death from cancer across races and ethnicities in the US^1^. In fact, breast cancer is the leading cause of death among U.S. Hispanic women, surpassing heart disease over a decade ago^2^.

Physical activity is recommended for most cancer patients as a nonpharmacological therapy to improve prognosis^3^. According to guidelines for cancer patients and survivors from American College of Sports Medicine, cancer survivors are suggested to complete 150 min/week of aerobic exercise and to perform strength training activities twice/week at moderate to vigorous intensity in order to ease cancer-related symptoms, improve general health and quality of life^4^. Studies have suggested that being physically active has a positive effect on physical and psychological levels: the underlying mechanisms might include regulation of sex hormones, metabolic hormones, adipokines, oxidative stress, and immune function^5^. Among women with breast cancer, there is accumulating epidemiological evidence indicating that survivors participating in physical activity of moderate to vigorous intensity have reduced all-cause mortality, breast cancer-specific mortality, risk of cancer-related symptoms, and improved physical health-related quality of life after their breast cancer diagnosis^6,7^. A systematic review by Lahart and colleagues^8^ found significant risk reductions for all-cause and breast cancer-related death for higher level of lifetime prediagnosis recreational physical activity (hazard ratio (HR) 0.82, 95% confidence interval (CI) 0.70–0.96, and HR 0.73, 95% CI 0.54–0.98, respectively), higher level of more recent prediagnosis physical activity (HR 0.73, 95% CI 0.65–0.82; and HR 0.84, 95% CI 0.73–0.97, respectively), and higher level of postdiagnosis physical activity (HR 0.52, 95% CI 0.43–0.64; and HR 0.59, 95% CI 0.45–0.78, respectively).

While most epidemiological studies including breast cancer patients have focused on the impact of specific physical activities, few have investigated the interaction of physical activities and other demographic or modifiable risk factors of death among women diagnosed with breast cancer. Factors of interest that could be important to the relationship between physical activity and survival among breast cancer patients due to their biological roles in energy balance are obesity, history of type 2 diabetes, and glucose level. Studies have found that breast cancer patients with diabetes experience a higher risk of breast cancer-specific and all-cause mortality, and being obese increases the risk of breast cancer-specific mortality by 45–57%^9–11^. We previously reported that fructosamine, a biomarker reflecting individual blood glucose level and closely related to diabetes, is negatively associated with breast cancer survival^12^. Furthermore, several recent studies have found that Hispanic ethnicity may modify the association of obesity, diabetes, and fructosamine level with mortality among breast cancer patients^12–14^, but the interaction between physical activity and ethnicity is not well established.

A previous study from the Health, Eating, Activity, and Lifestyle (HEAL) study reported that higher level of postdiagnosis moderate-intensity sports/recreational physical activity was associated with reduced risk of all-cause and breast cancer-specific mortality among breast cancer survivors, and that risk differed among different subgroups for age at diagnosis, ethnicity, body mass index (BMI), cancer stage, treatment, and ER status^15^. The present study provides an update to this previous analysis with longer follow-up time and examines a different timing of physical activity at approximately 5 months postdiagnosis. We hypothesize that this time window within months of diagnosis and the completion of treatment could serve as a critical timepoint for intervention to reduce risk of poor outcomes long-term. We also assessed the potential interactions between physical activity and other risk factors associated with all-cause mortality, breast cancer-specific mortality and non-breast cancer mortality, and our analysis is specific to the New Mexico HEAL site.

## Results

### Characteristics of study population

Table 1 presents the distribution of demographic and clinical characteristics of the study population by ethnicity, along with physical activity level. Among the 397 participants, a total of 133 deaths occurred during the median follow-up time of 13.5 years, of which 53 were breast cancer-specific deaths, and 80 were non-breast cancer-specific deaths. The median age at diagnosis of the study population was 58, while Hispanic women were significantly younger at diagnosis (54.83 ± 11.77) than non-Hispanic White women (60.07 ± 12.44). About 50% of participants had a BMI over 25 kg/m^2^ or were overweight. The mean BMI of Hispanic women was 27.21 ± 5.83 kg/m^2^, which was significantly higher than the mean BMI of 25.68 ± 5.36 kg/m^2^ for non-Hispanic White women. In addition, more Hispanic women received chemotherapy, while more non-Hispanic White received surgery and radiation. Regarding physical activity level, Hispanic and non-Hispanic White women reported a similar level of total physical activity (52.24 ± 38.18 MET-h/week for Hispanics and 46.05 ± 36.97 MET-h/week for non-Hispanic whites), moderate to vigorous physical activity (7.02 ± 12.24 MET-h/week for Hispanics and 9.20 ± 16.06 MET-h/week for non-Hispanic whites) and recreational activity (7.34 ± 12.36 MET-h/week for Hispanics and 9.59 ± 16.45 MET-h/week for non-Hispanic whites). However, Hispanic women tended to have higher household physical activity of 42.03 MET-h/week compared to non-Hispanic White women (33.90 MET-h/week).

**Table 1:** Baseline biological and clinical characteristics of the New Mexico HEAL study by ethnicity.

	Hispanic (*N* = 96)			Non-Hispanic White (*N* = 301)			
	*N*	%	Mean ± SD	*N*	%	Mean ± SD	*p*-value^#^
Characteristic							
Age	96		54.83 ± 11.77	301		60.07 ± 12.44	<0.01
BMI (kg/m^2^)	95		27.21 ± 5.83	298		25.68 ± 5.36	<0.01
Education							<0.01
8th grade or less	5	5.21		5	1.66		
Some high school	6	6.25		6	1.99		
High school grad	37	38.54		66	21.93		
Some college/tech	30	31.25		90	29.9		
College graduate	9	9.38		63	20.93		
Graduate school	9	9.38		71	23.59		
Tamoxifen use							0.12
No	57	59.38		162	53.82		
Yes	37	38.54		138	45.85		
Don’t know	2	2.08		1	0.33		
Fructosamine (μmol/L)							0.69
≤233 μmol/L	55	57.29		148	49.17		
>233 μmol/L	41	42.71		153	50.83		
			235.70 ± 41.12			237.24 ± 30.37	
Diabetes							0.20
No	85	0.8854		279	0.9269		
Yes	11	0.1146		22	0.0731		
Cancer Stage							0.61
Localized only	71	73.96		234	77.74		
Regional, NOS	25	26.04		66	21.93		
Unknown	0	0		1	0.33		
Treatment							0.047
Any chemotherapy	36	37.5		80	26.58		
Surgery and radiation	31	32.29		137	45.51		
Surgery only	29	30.21		84	27.91		
Physical activity (MET-h/week)^¶^							
Total	96		52.24 ± 38.18	301		46.05 ± 36.97	0.16
Moderate to vigorous	96		7.02 ± 12.24	301		9.20 ± 16.06	0.22
Vigorous	96		1.79 ± 8.10	301		2.91 ± 8.95	0.27
Household	96		42.03 ± 37.64	301		33.90 ± 31.90	0.04
Recreational	96		7.34 ± 12.36	301		9.59 ± 16.45	0.22

^¶^Physical activities reported during the past month (approximately 5 months post-breast cancer diagnosis) were categorized with different methods. By type, physical activities were categorized as household and recreational. By intensity, physical activities were categorized as moderate to vigorous (activities equivalent to >3 METs) and vigorous (activities equivalent to >6METs).

^#^*p* values were calculated to compare across ethnic groups. For continuous variables including age, BMI, fructosamine level, and physical activity levels, the *t*-test was applied; for categorical variables including education, tamoxifen use, diabetes, cancer stage and treatment, the *χ*^2^ test was applied.

### Associations between physical activity and mortality outcomes

Table 2 presents the age-adjusted and multivariable-adjusted hazard ratios (HRs) of all-cause mortality, breast cancer-specific mortality, and non-breast cancer mortality of different moderate to vigorous physical activity by ethnicity. Estimates of HRs from both age-adjusted and multivariable-adjusted models were similar. In fully adjusted models, women who participated in over 6.9 MET-h/week of moderate to vigorous physical activity at diagnosis had a 34% lower risk of death from any cause (HR 0.66, 95% CI 0.43–0.99, *p*-trend 0.04) than people who participated in no moderate to vigorous physical activity. The protective association was much stronger for non-Hispanic White women (HR 0.58, 95% CI 0.36–0.94, *p*-trend 0.02) than Hispanic women (HR 0.80, 95% CI 0.32–2.00, *p*-trend 0.64). A non-significant trend based on HRs of reduced risk was also observed for breast cancer-specific mortality (HR 0.70, 95% CI 0.36–1.40, *p*-trend 0.34) and non-breast cancer-specific mortality (HR 0.61, 95% CI 0.36–1.05, *p*-trend 0.05).

**Table 2:** Associations between mortality outcomes and moderate to vigorous physical activity (MET-hours per week) during the past month, overall and by ethnicity.

	Overall (*N* = 397)				Hispanic (*N* = 96)				NonHispanic White (*N* = 301)			
Outcome	0	>0–6.9	>6.9	*p*-trend	0	>0–6.9	>6.9	*p*-trend	0	>0–6.9	>6.9	*p*-trend
*All-causes death*												
No. of deaths/No. of participants	74/182	24/82	35/133		17/49	5/16	8/31		57/133	19/66	27/102	
*Model 1*^§^												
HR	1	0.76	0.64	0.02	1	1.09	0.78	0.58	1	0.66	0.57	0.01
95% CI		(0.48, 1.20)	(0.42, 0.95)			(0.38, 3.14)	(0.33, 1.94)			(0.39, 1.11)	(0.36, 0.90)	
*Model 2*^¶^												
HR	1	0.72	0.66	0.04	1	0.99	0.80	0.64	1	0.65	0.58	0.02
95% CI		(0.45, 1.16)	(0.43, 0.99)			(0.32, 3.06)	(0.32, 2.00)			(0.38, 1.09)	(0.36, 0.94)	
*Breast cancer death*												
No. of deaths/No. of participants	27/182	13/82	13/133		7/49	4/16	5/31		20/133	9/66	8/102	
*Model 1*^§^												
HR	1	1.01	0.60	0.14	1	1.41	0.95	0.97	1	0.88	0.47	0.08
95% CI		(0.52, 1.96)	(0.31, 1.16)			(0.41, 4.89)	(0.30, 3.02)			(0.40, 1.93)	(0.21, 1.08)	
*Model 2*^¶^												
HR	1	0.99	0.70	0.34	1	1.48	0.89	0.86	1	0.88	0.6	0.26
95%CI		(0.50, 1.95)	(0.36, 1.40)			(0.39, 5.67)	(0.27, 2.98)			(0.39, 1.96)	(0.25, 1.43)	
*Non-breast cancer death*												
No. of deaths/No. of participants	47/182	11/82	22/133		10/49	1/16	3/31		37/133	10/66	19/102	
*Model 1*^§^												
HR	1	0.59	0.64	0.06	1	0.75	0.74	0.66	1	0.53	0.57	0.04
95% CI		(0.30, 1.13)	(0.38, 1.06)			(0.08, 6.97)	(0.18, 2.95)			(0.26, 1.06)	(0.33, 0.99)	
*Model 2*^**¶**^												
HR	1	0.56	0.61	0.05	1	0.63	1.05	0.97	1	0.54	0.56	0.04
95% CI		(0.28, 1.09)	(0.36, 1.05)			(0.05, 6.53)	(0.22, 4.81)			(0.27, 1.09)	(0.31, 0.99)	

^§^Models adjusted for age and ethnicity (among all women) only.

^¶^Models adjusted for age, disease stage at baseline, treatment, BMI, diabetes, fructosamine, and ethnicity (among all women).

The protective associations between physical activity level and mortality for total, vigorous only and recreational physical activity by ethnicity were similar to those for moderate to vigorous physical activity (data not shown), while household physical activity showed a different pattern. As presented in Table 3, although none of the HR estimates were statistically significant, Hispanic women with the highest level of household activity had a higher magnitude of reduced risk of breast cancer-specific mortality (HR 0.41, 95% CI 0.17–1.01, *p*-trend 0.08) than the non-Hispanic White women (HR 0.80, 95% CI 0.50–1.29, *p*-trend 0.34).

**Table 3:** Associations between mortality outcomes and household physical activity (MET-hours per week) during the past month, overall and by ethnicity.

	Overall (*N* = 397)				Hispanic (*N* = 96)				Non-Hispanic White (*N* = 301)			
Outcome	<18	18–40.5	>40.5	*p*-trend	<18	18–40.5	>40.5	*p*-trend	0	>0–6.9	>6.9	*p*-trend
*All-cause death*												
No. of deaths/No. of participants	56/148	37/123	40/126		12/32	8/26	10/38		44/116	29/97	30/88	
*Model 1*^§^												
HR	1	0.73	0.77	0.18	1	0.66	0.59	0.23	1	0.76	0.81	0.34
95% CI		(0.48, 1.11)	(0.51, 1.15)			(0.27, 1.65)	(0.26, 1.37)			(0.47, 1.21)	(0.51, 1.29)	
*Model 2*^¶^												
HR	1	0.72	0.76	0.17	1	0.32	0.41	0.08	1	0.78	0.80	0.34
95% CI		(0.47, 1.10)	(0.50, 1.14)			(0.11, 0.95)	(0.17, 1.01)			(0.48, 1.27)	(0.50, 1.29)	
*Breast cancer death*												
No. of deaths/No. of participants	23/148	13/123	17/126		8/32	3/26	5/38		15/116	10/97	12/88	
*Model 1*^§^												
HR	1	0.66	0.85	0.57	1	0.46	0.49	0.2	1	0.77	1.04	0.06
95% CI		(0.33, 1.31)	(0.45, 1.60)			(0.12, 1.76)	(0.16, 1.50)			(0.35, 1.72)	(0.49, 2.24)	
*Model 2*^¶^												
HR	1	0.63	0.82	0.50	1	0.29	0.41	0.14	1	0.76	1.05	0.08
95%CI		(0.32, 1.26)	(0.43, 1.55)			(0.06, 1.34)	(0.12, 1.34)			(0.33, 1.74)	(0.49, 2.25)	
*Non-breast cancer death*												
No. of deaths/No. of participants	33/148	24/123	23/126		4/32	5/26	5/38		29/116	19/97	18/88	
*Model 1*^§^												
HR	1	0.83	0.8	0.39	1	0.94	0.89	0.87	1	0.83	0.78	0.40
95% CI		(0.49, 1.41)	(0.47, 1.36)			(0.24, 3.63)	(0.24, 3.34)			(0.46, 1.49)	(0.43, 1.42)	
*Model 2*^¶^												
HR	1	0.85	0.79	0.40	1	0.50	0.62	0.67	1	0.85	0.74	0.33
95% CI		(0.49, 1.47)	(0.46, 1.37)			(0.09, 2.73)	(0.13, 2.85)			(0.46, 1.57)	(0.40, 1.36)	

^§^Models adjusted for age and ethnicity (among all women) only.

^¶^Models adjusted for age, disease stage at baseline, treatment, BMI, diabetes, fructosamine, and ethnicity (among all women).

### Associations between physical activity and mortality by biological and clinical characteristics

**Fig. 1:**
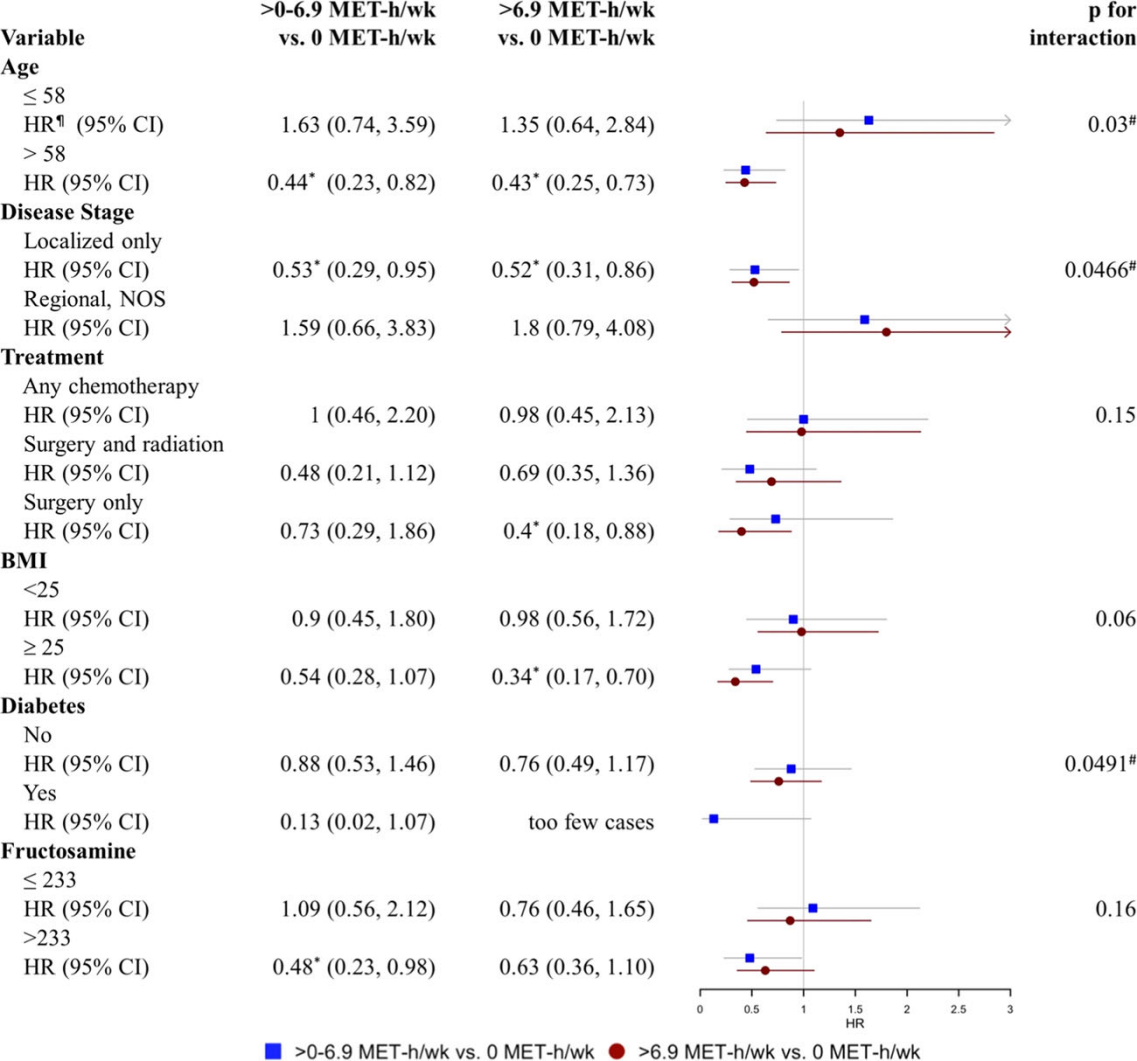
Associations between all-cause mortality and physical activity during the past month stratified by biological and clinical factors. The blue square and lines denotes HRs and 95% CIs for 0-6.9 MET hours/week compared to the reference group of 0 MET hours/week. The red circle andlines denotes HRs for greater than 6.9 MET hours/week compared to the reference group. The p values for statistical interactions are included for each variable. ¶ Models adjusted for age, disease stage at baseline, treatment, BMI, diabetes, fructosamine and ethnicity, * *p* < 0.05, # p for the interaction term < 0.05, indicating the statistically significant difference of the impact of moderate to vigorous physical activity on survival across subgroups.

Figure 1 presents the multivariable-adjusted HRs of all-cause mortality of the total study population stratified by biological and clinical characteristics including age at diagnosis, cancer stage, treatment, BMI, diabetes, and fructosamine level. The estimates within each subgroup for different physical activity types were similar, thus only HRs for moderate to vigorous physical activity are presented. Stronger protective associations were observed in certain subgroups. Women aged over 58 years old who participated in both moderate to vigorous levels of physical activity at diagnosis had reduced risk of all-cause mortality by 56% (HR 0.44, 95% CI 0.23–0.82) for 0 to 6.9 MET-h/week group and 57% (HR 0.43, 95% CI 0.25–0.73) for more than 6.9 MET-h/week group respectively, while the reductions in risk were not significant among women ≤58 years. The protective effect of participating in over 6.9 MET-h/week of moderate to vigorous physical activity was significantly stronger among women who were overweight (HR 0.34, 95% CI 0.17–0.70) than normal-weight (HR 0.98, 95% CI 0.56–1.72). Physically active women diagnosed with localized breast cancer had HRs of 0.44 (95% CI 0.23–0.82) and 0.43 (95% CI 0.25–0.73) respectively for the two active levels. However, physically active women diagnosed with regional cancer had increasing risk of all-cause mortality (HR 1.80, 95% CI 0.79, 4.08; HR 1.59, 95% CI 0.66, 3.83, respectively for the two physical activity levels), although not statistically significant.

We also observed larger benefits of being physically active in women treated with surgery only comparing to women with other treatment, and in women with fructosamine levels higher than 233 μmol/L compared to women with lower fructosamine levels, although interaction effects were not statistically significant. Our study lacked sufficient statistical power to evaluate differences by history of diabetes since only 10% of women reported having a history of diabetes. We also evaluated the interaction between tamoxifen use and physical activity, but no significant differences were observed (data not shown). Lastly, we evaluated the interaction effects between these variables and physical activity levels with risk of breast cancer-specific and non-breast cancer mortality. Similar trends of stronger protective associations were observed among older women, those with localized disease, and overweight women for both breast cancer-specific and non-breast cancer mortality (data not shown). However, these models were limited in statistical power due to the small number of deaths for each stratified group.

## Discussion

Our study results suggest that breast cancer survivors who participated in higher physical activity levels within several months of diagnosis have a reduced risk of all-cause mortality, even after 13 years of follow-up. Furthermore, this protective association differs by physical activity type. The protective association between higher moderate to vigorous physical activity level with all-cause mortality was observed in the non-Hispanic White population, but was not apparent among the Hispanic population. Though most of the associations with household physical activity were not statistically significant, Hispanic women with the highest level of household activity had more of a reduced risk of breast cancer-specific mortality than non-Hispanic White women. Additionally, biological and clinical characteristics including age at diagnosis and disease stage were found to significantly alter the effect of physical activity on the risk of survival after breast cancer.

Several aspects of our findings were similar to the previous HEAL report results regarding the protective association of higher physical activity level on survival, although the time of assessment of physical activity level was different^15^. The previous study focused on prediagnosis (1 year before) and postdiagnosis (3 years after) physical activity, while we examined physical activity around the time of diagnosis (approximately 5 months after). In the HEAL study population, participants’ physical activity level was found to be reduced significantly after breast cancer diagnosis^16^. Our study results provide insight from the perspective of how being physically active shortly after diagnosis affects long-term mortality among breast cancer survivors. Compared to the previous study, we had a smaller study population as we only included the New Mexico site of the HEAL study (397 vs. 688), but we had a longer median follow-up time (13.5 years from baseline vs. 6 years from baseline) and more cases of death (133 deaths vs. 53 deaths), which allowed us to better evaluate the long-term effect of physical activity. While our overall results were similar to the prior HEAL report, their stratified analyses were not statistically different by age, BMI, disease stage, treatment, or ER status. We however observed significantly stronger protective associations in older breast cancer survivors and those with localized cancer.

Additionally, we analyzed whether physical activity levels influenced non-breast cancer mortality. We found an inverse trend between physical activity and non-breast cancer deaths, and the association was significant among non-Hispanic White women. Since a large proportion of the non-breast cancer deaths in our study population were deaths due to cardiovascular disease (CVD), our results are in agreement with other studies that reported the benefit of physical training on CVD mortality among the general population^17,18^. Although this association among breast cancer survivors has not been well established, a few preclinical studies have suggested physical exercise can reduce doxorubicin-related cardiotoxicity introduced during breast cancer treatment and thus improve cardiovascular function in animal models^19–21^. There are also studies showing that cardiovascular risk factors including overweight, hypertension and hyperlipidemia can be controlled after the implementation of exercise intervention among breast cancer survivors ^22–24^.

To our knowledge, few studies have evaluated the association of physical activity and mortality in the U.S. Hispanic population. Our findings are different from those of the New Mexico Women’s Health Study (NMWHS)^7^, where our colleagues suggested a significant reduction of all-cause mortality in Hispanic breast cancer survivors only. Differences in assessing and categorizing physical activity and the case–control design of the NMWHS may account for the differences in findings. However, although the magnitude and significance levels were different, the reduction trends were similar. Another possible reason is we did not observe this significant reduction in risk among Hispanic women might be due to the small study population and limited statistical power to detect a difference in mortality within this ethnic group.

The differences observed for the magnitude of associations by ethnicity in our study might also be partially explained by the difference in physical activity patterns and other characteristics across ethnicity. In our study population, though Hispanics and non-Hispanic White women reported similar total physical activity patterns, the Hispanic population had slightly lower moderate to vigorous recreational physical activity levels and significantly higher household activity levels. Hispanic women were also younger and more likely to be overweight or obese than non-Hispanic White women in our study. The characteristics of Hispanic women in our study were similar to the New Mexico breast cancer population in other studies. NMWHS reported that household activities took up 62.2% of total activity in Hispanic women and 52.9% in non-Hispanic White women^7^. Hispanic women are also considered to have a higher prevalence of overweight and obesity compared to non-Hispanic Whites in the general population^25^.

Although the effects of physical activity on certain subgroups of breast cancer survivors has not been well established, some studies have suggested that there is a reduction in the risk of mortality outcomes among active older patients/survivors and among women who are physically active and overweight^26^. We observed a stronger protective effect among the older women compared to younger women and among overweight women compared to the normal-weight women. In a Nurses’ Health Study analysis conducted by Holmes et al.^27^, postmenopausal women who were physically active (with equal or more than nine MET-h/week) had a significant reduction in risk of breast cancer mortality (HR: 0.73, 95% CI 0.54–0.98) while the reduction was not significant among the premenopausal women (HR: 0.73, 95% CI 0.54–0.98). Regarding different BMI groups, studies by Cleveland et al.^28^ and Holick et al.^29^ both showed nonsignificant lower breast cancer-specific mortality in active normal-weight patients (BMI < 25) and significantly lower risk in active overweight (BMI ≥ 25) patients.

A major strength of this study is that we evaluated the difference in response to physical activity among ethnically diverse breast cancer survivors by specific subgroups, including women over the age of 58 years at breast cancer diagnosis, women who were overweight, and women diagnosed with localized disease; all of whom might benefit more from physical activity interventions after their breast cancer diagnosis. While older and overweight patients^26^ and Hispanic women^30,31^ generally have a worse prognosis after breast cancer diagnosis and treatment, these study results may add to the development of personalized physical activity recommendations following a breast cancer diagnosis.

Some limitations should be considered when interpreting the study results. The primary limitation is the small sample size. Compared to a similar study evaluating the association between physical activity and survival among breast cancer survivors by ethnicity, NMWHS (722 participants)^7^, we had a smaller study population. Our smaller study may have limited our power to observe and interpret the true association. However, we had an average follow-up time of 13.5 years, which was comparable to the 14.7 years follow-up in NMWHS. The small sample size also limited our power to conduct stratified analysis for breast cancer-specific death and non-breast cancer death. An additional limitation of our study design was that we were unable to assess to changes in physical activity levels throughout follow-up. Other limitations are recall bias and information bias introduced by self-reported physical activity. Studies have shown, in relation to social desirability bias and other possible theories, study participants have been known to over-report physical activity behavior, and this overestimation was found to be greater in the older population and higher BMI groups^32–35^. Self-reported information was not assessed using other methods in the HEAL study to address the reporting bias. However, we did not observe significantly higher overall physical activity patterns in the older group or the overweight group.

In conclusion, being physically active at the time of a breast cancer diagnosis can improve prognosis by reducing the risk of all-cause mortality. Furthermore, specific subgroups with certain biological and clinical characteristics might benefit even more from physical activity. Although the present study had limited power in making precise interpretations for other causes of death, our study findings can still inform the public health implications of physical activity interventions or specific recommendations for breast cancer survivors, particularly for certain subgroups who might be at higher risk of poor outcomes.

## Methods

### Study population

The New Mexico HEAL study is part of a multicenter, multiethnic prospective cohort study designed to examine the associations of various modifiable and nonmodifiable factors with prognosis among women diagnosed with in situ to Stage IIIA breast cancer. Detailed study objectives, eligibility, recruitment, data collection and quality control procedures are described elsewhere^16,36,37^. Briefly, a total of 999 women with primary breast cancer, aged 18 years or older, diagnosed with in situ to Stage IIIA primary breast cancer between July 1996 and March 1999 living Bernalillo, Santa Fe, Sandoval, Valencia, or Taos counties were ascertained through the Surveillance, Epidemiology, and End Results (SEER) registry in New Mexico. Of these, 615 women participated. Participants were asked to complete three self-reported health and medical assessments within 2 years after diagnosis and a voluntary 50 mL blood draw.

### Ethics

Written informed consent was obtained from all study participants. The study was approved by the Human Research Protections Office at the University of New Mexico, in addition to the Institutional Review Board for Human Subjects at the University of Louisville.

Data for the present study was derived from the in-person interview of the New Mexico HEAL study at baseline (5.7 ± 1.7 months postdiagnosis) and the New Mexico Tumor Registry (NMTR) records. Among the 615 participants, 216 women were excluded because of having in situ breast cancer (*n* = 99) and the lack of a stored plasma sample collected at baseline interview (*n* = 117). We further excluded women who had no information on date of interview (*n* = 2). The final analyses include a total of 397 invasive breast cancer survivors, among which 96 were Hispanic and 301 were non-Hispanic White.

### Physical activity assessment

Physical activity, including recreational, household and occupational activities, were collected using a questionnaire based on the Modifiable Activity Questionnaire developed by Kriska et al., which has been shown to be valid and reliable^38,39^. During the baseline interview, study participants were asked by interviewers about their activity types, frequency, and duration at two timepoints: one year prior to diagnosis and in the past month. For the present analysis, physical activity reported during the past month, which was approximately 5 months after their breast cancer diagnosis, was the primary exposure.

A metabolic equivalent of task (MET) value was assigned to each activity based on the revised Compendium of Physical Activity^40^. In the guideline^40^, sitting is equivalent to 1.0 MET, walking in different speed with carrying things is equivalent to METs ranging from 2.0 to 3.8, and running in varying condition is equivalent to METs ranging from 8.0 to 15.0. Physical activities were categorized by types as household activities and recreational activities, and by intensiveness as moderate to vigorous activities (>3 METs) and vigorous only activities (>6 METs)^40^. Weekly average energy expenditure for each physical activity was measured by MET-hours per week (MET-h/wek), which was computed by MET value times weekly frequency and duration.

### Covariates

Stage of disease (local I, regional(II/IIIa) or unknown) and breast cancer treatment (any chemotherapy, surgery and radiation, or surgery only) were obtained from medical record abstraction and NMTR registry records. Height and weight were measured and BMI was computed as weight (kg) divided by the square of height (m). Waist and hip circumferences were measured at the clinic visit to compute a waist-hip ratio. Aliquots from stored plasma samples were used for fructosamine measurement. Plasma fructosamine level was measured at the Rifai Laboratory of Boston Children’s hospital on the Roche P Modular system using product from Roche^12^. Demographic information and other covariates, including age at breast cancer diagnosis, education, ethnicity (nonHispanic White and Hispanic), tamoxifen use and self-reported diabetes history, were assessed at during an interviewer-administered questionnaire.

### Outcome assessment

Participants’ vital status information was obtained through linkage with NMTR from the National Death Index records. Participants were followed by NMTR till death or December 2013, whichever occurred first, with a median follow-up time of 13.5 years. Survival time was defined as from the date of interview to death or December 2013. Breast cancer-specific mortality was calculated using breast-cancer specific death, which was classified as died from breast cancer based on the International Classification of Diseases, 10th revision (ICD-10) code C50^41^. All deaths with a cause of death other than breast cancer were considered to be non-breast cancer specific.

### Statistical analysis

Means and standard deviations (SDs) were calculated to describe the demographic and clinical characteristics of all participants by ethnicity. *p*-values were calculated by chi-square tests and *t*-tests to compare across ethnic groups. Cox proportional hazards models were applied to compute hazard ratios (HRs) for death classified as all cause, breast cancer-specific, and non-breast cancer-specific mortality by ethnicity. Total physical activity, moderate to vigorous physical activity, household physical activity, and recreational physical activity were evaluated in tertiles based on distribution within each stratum. Vigorous only activities were evaluated as binary (no vigorous activity vs. some vigorous activity) due to the fact that only a few participants (*n* = 72) had a MET-h/week greater than zero for vigorour activity. The group with the lowest active time was considered to be the reference group in each stratum. Aside from household activities, results were similar for other types of physical activity. Thus, only results for household activities and moderate to vigorous activities are presented.

No covariates other than age altered the HRs by 10%, thus covariates were retained if considered to be biologically meaningful based on previous literature^7,15,29^. Waist-hip ratio was highly correlated with BMI, so we included BMI only. The final models included one age-adjusted model and a multivariable model adjusting for age at diagnosis, BMI, breast cancer treatment, stage of disease, self-reported diabetes, and plasma fructosamine. The proportional hazard assumption was examined by interaction between physical activity and other covariates with time. None of the interactions were significant, therefore the assumption was not violated. Linear trends were tested using the Wald test by treating exposure tertiles as ordinal.

To assess the interaction between physical activity and other risk factors of death among participants, we also applied multivariable cox proportional hazard models stratified by each risk factor of interest adjusting for remaining covariates. *p*-values of interaction terms of risk factors and physical activity measures were presented. All analyses were performed using Stata/SE Version 15.0 (StataCorp, College Station TX) and SAS version 9.4 (SAS Institute, Cary NC) with significance level of 0.05.

### Reporting summary

Further information on experimental design is available in the Nature Research Reporting Summary linked to this paper.

## Supplementary information

Reporting Summary

## Data Availability

The data generated and analysed during this study are described in the following data record: 10.6084/m9.figshare.12918392^42^. The data generated and/or analysed during this study are contained in a single SAS (Statistical Analysis Software) file called “HEAL_Invasive_Data.sas7bdat”. The data are not publicly available as they contain information that could compromise research participant privacy. However, the data can be made available upon reasonable request and subject to an appropriate privacy agreement. Data requests should be made to the senior author, Richard N. Baumgartner.
