# Supplementary material for: Ethnic and biological differences in the association between physical activity and survival after breast cancer
Source: NPJ Breast Cancer. 2020 Oct 9;6:51. doi: 10.1038/s41523-020-00194-5 (PMC7547070; doi:10.1038/s41523-020-00194-5)
Supplement: Supplementary file 1 — Reporting Summary [file 41523_2020_194_MOESM1_ESM.pdf]

nature research

Corresponding author(s): Avonne Connor

Last updated by author(s): 09/04/2020

Reporting Summary

Nature Research wishes to improve the reproducibility of the work that we publish. This form provides structure for consistency and transparency in reporting. For further information on Nature Research policies, see Authors & Referees and the Editorial Policy Checklist.

Statistics

For all statistical analyses, confirm that the following items are present in the figure legend, table legend, main text, or Methods section.

n/a	Confirmed	
☐	☒	The exact sample size (n) for each experimental group/condition, given as a discrete number and unit of measurement
☐	☒	A statement on whether measurements were taken from distinct samples or whether the same sample was measured repeatedly
☐	☒	The statistical test(s) used AND whether they are one- or two-sided<br>*Only common tests should be described solely by name; describe more complex techniques in the Methods section.*
☐	☒	A description of all covariates tested
☐	☒	A description of any assumptions or corrections, such as tests of normality and adjustment for multiple comparisons
☐	☒	A full description of the statistical parameters including central tendency (e.g. means) or other basic estimates (e.g. regression coefficient) AND variation (e.g. standard deviation) or associated estimates of uncertainty (e.g. confidence intervals)
☐	☒	For null hypothesis testing, the test statistic (e.g. F, t, r) with confidence intervals, effect sizes, degrees of freedom and P value noted<br>*Give P values as exact values whenever suitable.*
☒	☐	For Bayesian analysis, information on the choice of priors and Markov chain Monte Carlo settings
☒	☐	For hierarchical and complex designs, identification of the appropriate level for tests and full reporting of outcomes
☐	☒	Estimates of effect sizes (e.g. Cohen's d, Pearson's r), indicating how they were calculated

Our web collection on statistics for biologists contains articles on many of the points above.

Software and code

Policy information about availability of computer code

Data collection	after data collection was performed (in person by trained study interviewers or by the New Mexico SEER tumor registry medical abstractors), data was stored in SAS data sets.
Data analysis	Stata/SE version 15.0 and SAS version 9.4

For manuscripts utilizing custom algorithms or software that are central to the research but not yet described in published literature, software must be made available to editors/reviewers. We strongly encourage code deposition in a community repository (e.g. GitHub). See the Nature Research guidelines for submitting code & software for further information.

Data

Policy information about availability of data

All manuscripts must include a data availability statement. This statement should provide the following information, where applicable:

- Accession codes, unique identifiers, or web links for publicly available datasets
- A list of figures that have associated raw data
- A description of any restrictions on data availability

The data that support the findings of this study are available on request from the senior author, (R.N. B.). The data are not publicly available due to restrictions with them containing information that could compromise research participant privacy.

Field-specific reporting

Please select the one below that is the best fit for your research. If you are not sure, read the appropriate sections before making your selection.

☐ Life sciences ☒ Behavioural & social sciences ☐ Ecological, evolutionary & environmental sciences

For a reference copy of the document with all sections, see nature.com/documents/nr-reporting-summary-flat.pdf

Behavioural & social sciences study design

All studies must disclose on these points even when the disclosure is negative.

Study description	The New Mexico HEAL Study is a prospective case-cohort study conducted between 1996 and 1999. (quantitative study)
Research sample	A total of 615 study participants, 18 years of age or older, diagnosed with first primary breast cancer (in situ to stage IIIA) between July 1996 and March 1999 were included in the parent study. Participants were residents of Bernalillo, Santa Fe, Sandoval, Valencia, or Taos counties at the time of diagnosis. Subjects who were diagnosed with invasive breast cancer (stages I-IIIA) and with a stored baseline plasma sample were eligible for inclusion in the present analysis (n=99 in situ cases excluded). A total of 397 invasive breast cancer survivors (96 Hispanic, 301NHW) were included in the present study.
Sampling strategy	A total of 999 eligible first primary breast cancer cases were ascertained through the National Cancer Institute's Surveillance Epidemiology and End Results (SEER) registry in New Mexico (sampling strategy convenience).
Data collection	Tumor characteristics and treatment information were abstracted from medical records. Baseline demographic characteristics, medical history, and lifestyle factors prior to diagnosis were collected approximately 5 months post-diagnosis by trained interviewers (interview packets were completed by the trained interviewer with pen and paper). A blood sample and anthropometric measurements were also collected at baseline interview. Ethnicity was based on self-report and was assessed at the time of screening for eligibility and at baseline interview.
Timing	Baseline data collection occurred approximately 5-6 months post-diagnosis.
Data exclusions	Exclusions included: Women without a stored plasma sample (n=117) and then those with in-situ breast cancer (n=99), women missing date of interview (n=2)
Non-participation	not applicable for the present analysis (secondary data analysis)
Randomization	No randomization. Covariates were adjusted in final models.

Reporting for specific materials, systems and methods

We require information from authors about some types of materials, experimental systems and methods used in many studies. Here, indicate whether each material, system or method listed is relevant to your study. If you are not sure if a list item applies to your research, read the appropriate section before selecting a response.

Materials & experimental systems

n/a	Involved in the study	
☒	☐	Antibodies
☒	☐	Eukaryotic cell lines
☒	☐	Palaeontology
☒	☐	Animals and other organisms
☐	☒	Human research participants
☒	☐	Clinical data

Methods

n/a	Involved in the study	
☒	☐	ChIP-seq
☒	☐	Flow cytometry
☒	☐	MRI-based neuroimaging

Human research participants

Policy information about studies involving human research participants

Population characteristics	See above
Recruitment	A total of 999 eligible first primary breast cancer cases were ascertained through the National Cancer Institute's Surveillance Epidemiology and End Results (SEER) registry in New Mexico. Participation rates were higher among NHWs (65%) compared to Hispanics (55%). A total of 615 study participants, 18 years of age or older, diagnosed with first primary breast cancer (in situ to stage IIIA) between July 1996 and March 1999 were included in the parent study.

Ethics oversight

The study was approved by the Human Research Protections Office at the University of New Mexico, in addition to the Institutional Review Board for Human Subjects at the University of Louisville

Note that full information on the approval of the study protocol must also be provided in the manuscript.
